# Air emissions and health risk assessment around abattoir facility

**DOI:** 10.1016/j.heliyon.2020.e04365

**Published:** 2020-07-12

**Authors:** Ebenezer Leke Odekanle, Omowonuola Olubukola Sonibare, Oludare Johnson Odejobi, Bamidele Sunday Fakinle, Funso Alaba Akeredolu

**Affiliations:** a Department of Chemical Engineering, Landmark University Omu-Aran, Kwara State, Nigeria; b Department of Community Health, College of Health Sciences, Obafemi Awolowo Univeristy, Ile Ife, Nigeria; c Department of Chemical Engineering, Obafemi Awolowo University, Ile Ife, Nigeria; d First Technical University, Ibadan, Oyo State, Nigeria

**Keywords:** Abattoir, Air quality, Particulate matter (PM), Air quality index, Dispersion, Cancer, Environmental chemical engineering, Atmospheric chemistry, Environmental analysis, Environmental health, Environmental impact assessment, Environmental risk assessment, Environmental science, Chemistry, Chemical engineering

## Abstract

The study assessed the impacts of abattoir activities on ambient air quality and health risk associated with exposure to PM_2.5_ and PM_10_, H_2_S, SO_2_ and NH_3_. Air samplings were done simultaneously around the abattoir at three points for sixty consecutive days (October to November) and standard methods adopted for the samplings and analysis. Health risks associated with exposure to PM_10_ and PM_2.5_ were estimated, using attributable fractions, relative risk and the excess lifetime cancer risk. The non-carcinogenic risks induced by the inhalation of H_2_S, SO_2_ and NH_3_ were also evaluated using hazard quotient (HQ). The results indicated that the average concentrations of 18.75 μg/m^3^, 89.17 μg/m^3^ and 0.1ppm for PM_2.5_, PM_10_ and NO_2_ respectively, were higher than the World Health Organization (WHO), National Ambient Air Quality Standard (NAAQS) and Federal Ministry of Environment (FMEnv) permissible limits. Air Quality Index showed that the ambient air quality in respect of CO and NH_3_ was very good, moderate for PM_10_ and was very poor for NO_2_ and SO_2_. It was also shown that 0.32% of deaths from lung cancer, and 0.23% from cardiopulmonary could be avoided if PM_2.5_ is reduced to 3 μg/m^3^ and while about 0.14% of all-cause mortality could be avoided if PM_10_ is reduced to 10 μg/m^3^. In similar manner, at least 0.45% likelihood that an individual in a group of people exposed to PM_2.5_ 100m away from the burning point may have health issue (lung cancer) than an individual from another set of people that is exposed to baseline concentration of 3 μg/m^3^. All the HQ values exceeded the threshold value, set at the unity, implying that H_2_S, SO_2_ and NH_3_ are likely to cause adverse health effects in the area. Conclusively, continuous operation of this abattoir within the residential area can constitute a great environmental menace to the residents of the area and can result in complication to those with existing health challenge.

## 1 Introduction

Air is said to be polluted when it is contaminated as a result of alteration of its natural composition either by natural occurrence or anthropogenic activities (Umunnakwe and Njoku, 2017). The contaminants such as dust, fumes gas, mist, odors, smoke or vapor could be present in the polluted atmosphere in such quantities, and for such period of time that make them injurious to human, plant or animal life or to property. When air is polluted either by the release of thick smoke emanating from burning or foul smell from heaps of waste materials, it does not only cause interference to the comfort of human beings but also adversely affect the lives of properties, plants and animals within the vicinity. Worldwide, abattoirs have been adjudged as one of the major sources of air pollution (WHO, 1987). Abattoirs’ impacts on the ambient air quality vary from being minor to major; depending on whether control measures are put in place or the emissions are allowed to constitute environmental nuisance and threat to public health (Auwalu et al., 2015).

In abattoir operation, apart from the problems associated with the handling of the animal wastes, the substantial amount thick black fume generated while the animals are being burnt often pollutes the air. Umunnakwe and Njoku (2017) reported that during the course of processing of the animals for human consumptions, the animals are roasted with kerosene and condemned tyres and this practice leads to the release of carbon monoxide (CO) into the environment. Physical inspection of many abattoirs in Nigeria revealed that the common practice of slaughterhouse operators involves open burning of the slaughtered animals and of heaps of dry abattoir wastes; and this could result into the release of both particulate matters and gaseous pollutants including volatile organic compounds into the atmosphere. Although none of the abattoirs visited employed the use of incinerator, its use could not be totally relied upon because emission from the incinerator could also constitute a source of serious worry to the residents around abattoir. Various unfriendly abattoir operations such as indifferent dumping and unhygienic discharge of abattoir waste effluent have been reported to be one of the factors responsible for the alteration of air quality of abattoir environment (Ubuoh et al., 2017). This pollution results in the unpleasant odour and consequentially, has unfavorable health implications on the residents especially on individuals with existing medical challenge. Magaji and Hassan (2017) equally revealed that gaseous pollutants around abattoir facility could exceed recommended limit, thereby making the air unhealthy for the people around the abattoir. Other studies have also opined that abattoir operations in developing nations pollute the environment directly or indirectly which may result into serious health problems (Olowoporoku, 2016; Adonu et al., 2017; Daramola and Olowoporoku, 2017; Adonu et al., 2017; Ubuoh et al., 2017). Studies have documented linkages between health effects and air pollution (Ghorani-Azam et al., 2016; ATSDR, 1998; Wang et al., 2018; Zhong et al., 2019). According to World Health Organization (WHO, 2018) exposure to air pollutants can lead to serious health effects ranging from respiratory related diseases to chronic diseases that could lead to high mortality. The nature of emission produced from abattoir operation introduces so much odoriferous compounds into the atmosphere and this in turn affects the air quality making it unbearable for human (EPA, 2001; ATSDR, 1998). Of interest is the emission of pollutants such as CO, Sulphur dioxide (SO_2_), Nitrogen dioxide (NO_2_) Volatile Organic Compounds (VOC) and Particulate Matter (PM) from a typical abattoir operation. High concentration of these pollutants can be objectionable and can result in health challenges such as nausea, headache, eye irritation, paralysis and even death. Irritations in the eye, nose and throat as well as loss of coordination are associated with exposure to pollutants such as SO_2_, NO_2_ and PM_10_, and VOCs (Ubuoh et al., 2017). Chronic lung infections among abattoir workers as well as respiratory track-infections among people living close to abattoir are linked to exposure to emission from abattoir (Ubuoh et al., 2017; Ekpo, 2019). In the long run, some of these are suspected to cause damage to the liver and other vital organs of the body which may even lead to death (Ubuoh et al., 2017). Children and aged adults are most vulnerable to these organic pollutants. Annual averages, according to WHO guideline for outdoor air quality for NO_2_, SO_2_, PM_10_ and PM_2.5_ are 40, 20, 20 and 10 μg/m^3^ respectively (WHO, 2018), while Nigerian Ambient Air Quality Standards (NAAQS) allowable limits for PM_10_, NO_2_, SO_2_, and H_2_S are 150 μg/m^3^, 0.03, 0.03 and 0.005 ppm respectively (FEPA, 1999). However, even the concentrations of air pollutants lower than these guidelines are also known to effect human health adversely (Gilbert et al., 2019). In spite of all these environmental havocs caused by improper abattoir operational system, the construction of abattoirs in Nigeria is always on the increase without concern for environmental impact of the abattoirs on the localities. Studies have been conducted on the appraisal of the effects of abattoir on water and land qualities (Bala et al., 2016; Ojekunle and Lateef., 2017; Daramola and Olowoporoku, 2017; ATSDR, 2014; Igbinosa and Uwidia, 2018; Elemile et al., 2019). Also air pollution health risk assessments methods have been reported (Kunzli et al., 2000) and subsequently employed in several air pollution projects across the globe (Wesson et al., 2010; Lim et al., 2012; Lopez, 2013; Adonu et al., 2017; Burnett et al., 2014; Zhong et al., 2019). Usually, health risk assessment of air pollution is carried out using single data to evaluate the risk (Edokpolo et al., 2015). However, the use of probabilistic approach has also been established to be suitable for the evaluation of variability of the risk (Edokpolo et al., 2014; Arranz et al., 2014) to provide a quantitative description of uncertainty and variability in assessing the health risk (Cao et al., 2011; ATSDR, 2004; ATSDR, 2014). Owing to the scarcity of limited reported studies on the effects of abattoir operations on ambient air quality and the associated health implications, it is crucial to assess the environmental impact and health risk associated to exposure to emissions from abattoir operations. Therefore, this study investigates exposure to emission from the abattoir relative to closeness to the operation point as well as the impacts of abattoir operations on ambient air quality and human health based on standard methods. This study will provide information on the magnitude of air pollution from abattoir and the need for reduction of dispersion of the air pollutants to residential areas.

## 2 Material and methods

### 2.1 Sampling site

**Figure 1:**
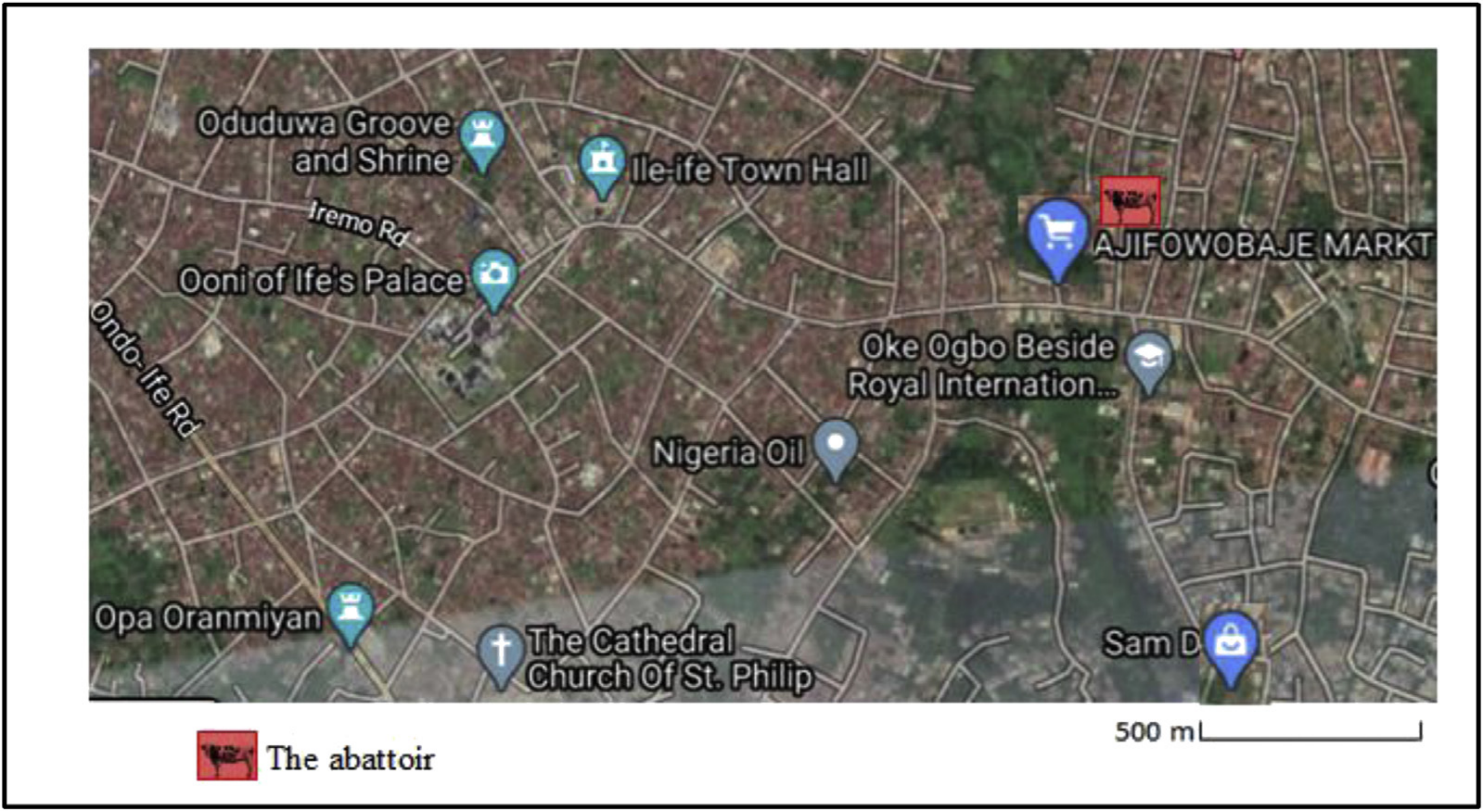
Map of the study area (google map).

The abattoir facility is located in Ile-Ife, one of the major cities in Southern region of Nigeria. The study area has two distinct climatic seasons: dry and wet seasons. Although variation may occur, dry season runs from November to February, while wet season spans March to October (Umunnakwe and Njoku, 2017). The choice of Ile Ife was based on some special criteria and feature the city has. Apart from being one of the cities in Southern Nigeria with high population density, it is also faced with environmental issue which has become worrisome to the residents. The choice of the abattoir was based on its size and location. The abattoir is the biggest in the city slaughtering average of twenty animals on daily basis. The mode of operation involves slaughtering and burning the animals in an open air with kerosene and subsequent discharge of the effluents into a nearby flowing river. Apart from the average of four hundred people that patronize the abattoir on daily basis, several restaurants and food joints depend on the abattoir for meat supply. The abattoir is not only located within residential area with population of about two thousand, but also adjacent to one of the major markets in the city (Figure 1).

### 2.2 Sampling equipment

Sampling of the particulate matter was done using Aerosol Mass Monitor (831, U.S.A) while concentrations of gaseous pollutants were measured using Aeroqual series (200, U.S.A). Aerosol Mass Monitor is a portable device which is capable of measuring simultaneously five mass ranges of particulates: PM_1_, PM_2.5_, PM_4_, PM_10_, and TSP in μg/m^3^ with a concentration range of 0–1 μg/m^3^, a sampling time of 4 min, a flow rate of 2.83 1/min. The device comes with an iso-kinetic probe that is attached to inlet nozzle. The probe helps reduce count errors related to the sample flow velocity and the aerodynamics of small particles. To measure, the monitor is first switched on in the environment of interest to stabilize for several minutes after which ‘START’ key is pressed to begin a four minutes cycle of sampling. When in operation, air is drawn in through a small optical orifice, and a laser optical system counts and sizes the particles as they pass through. The pulses from the detector are stored in one of the four memory banks and are converted into mass. A sound of internal vacuum pump indicates the end of a cycle which is then followed by pressing ‘SELECT’ key to display concentration in size ranges on the screen of the monitor. The monitor will display the result until the ‘START’ key is pressed to begin another cycle of sampling or until the unit is switched off. Any data accumulated is lost when the ‘STOP’ key is pressed. Also, Aeroqual series is a portable gaseous pollutants measuring device, having a strong built-in sampling pump that sucks up air vertically and horizontally up to about 100 feet (30 m). The monitor combines a PID (Photoionization Detector) with sampling pump having a detection range of 0–2000 ppm. This device uses Lithium ion battery and it is turned on at sampling to measure the concentration of pollutants of interest and the result is displayed on the screen of the equipment. The results remain on the screen until one presses a ‘start’ key to commence another round of sampling. To calibrate the device, the “enter” button is pressed and until the word “Zeroing”appears next to ZERO CAL. This routine always runs for up to ten minutes (depending on the gas sensor installed) and then the device beeps to indicate completion. During the entire study period, Kestrel 4000 pocket weather tracker was utilized to monitor temperature, relative humidity and wind speed.

### 2.3 Air sampling procedure

Air sampling focused mainly on SO_2_, NO_2_, PM_10_, PM_1_ and CO since these pollutants constitute large portion of emission from abattoir. Other pollutants measured include VOC, H_2_S and NH_3_. For comparative analysis, three air sampling locations were chosen: The upstream (point A), the discharge station (point B) and the downstream (point C). The discharged location is the point where the animal is being burnt and process. Upstream and downstream are 100 m before and after discharge point respectively. At the point of sampling, the devices (Aerosol Mass Monitor and Aeroqual series gas monitors) were turned on to measure the concentrations of both particulate matter and gaseous pollutants respectively. Sampling was done between 6:00 a.m and 2 p.m. (the abattoir operational period) on 8-hourly with one hour interval on daily basis for sixty consecutive days (from October to November), including Saturdays and Sundays when the abattoir was not in operation (non-work days) in order to further assess the contribution of the abattoir operation to the ambient air quality of the environment. All the measurements were done simultaneously at the three sampling points both on wet and dry days in order to assess the impact of humidity on the pollutant concentration levels. The data obtained were statistically analyzed and mean values were compared with standards and guideline as well as air quality index. Since the abattoir is far from road, the impact of vehicle emissions and other sources of air emission within the premises of the abattoir were not included in the investigation as this was not identified to be major source of pollution at the abattoir.

### 2.4 Health risk assessment

Health risk assessment of PM_10_, PM_2.5_, H_2_S, SO_2_ and NH_3_ were carried following the standard methods (EPA, 1997; Ostro, 2004; US EPA, 2009) and subsequently employed in similar studies (Kitwattanavong et al., 2013; Hyungkeun et al., 2018; Chalvatzaki et al., 2019; Gilbert et al., 2019). In this study, two methods adopted for the health risks associated with exposure to PM_10_ and PM_2.5_ were Environmental Burden Disease (EBD) due to air pollution (Chalvatzaki et al., 2019), and Excess lifetime Cancer Risk (Hyungkeun et al., 2018). Environmental Burden Disease involves using attributable fractions (AF) which estimates the proportion of deaths arising from a disease (e.g. lung cancer or cardiopulmonary mortality) which could be prevented if particulate matter levels were lowered to 10 μg/m^3^ and 3 μg/m^3^ for PM_10_ and PM_2.5_ respectively (Chalvatzaki et al., 2019) and relative risk (RR) which is an estimates of the probability that there would be occurrence of health implication (e.g all-cause mortality or lung cancer mortality) in a group of people exposed to PM_10_ higher than 10 μg/m^3^ (Ostro, 2004). Attributable fraction and relative risk were calculated using Eqs. (1), (2a), (2b) (Ostro, 2004; Chalvatzaki et al., 2019) respectively.

(1) $$\text{AF}=\frac{RR-1}{RR}$$

where the quantity RR-1 represent the excess risk, ER". RR for cardiopulmonary and lung cancer mortality associated with exposure to PM_2.5_ was obtained from

(2a) $$\text{RR}={\left[\left(\text{X}+1\right)/\left({\text{X}}_{\text{0}}+1\right)\right]}^{\text{β}}$$

X = mean concentration of the pollutants; X_o_ **=** baseline concentration: 10 μg/m^3^ for PM_10_ and 3 μg/m^3^ for PM_2.5_; β = coefficient of risk function for long term exposure (0.15515; 95% CI: 0.0562–0.2541) for cardiopulmonary mortality while for lung cancer mortality and 0.23218 (95% CI: 0.08563–0.37873) (Ostro, 2004; Chalvatzaki et al., 2019).

The relative risk (RR) due to all-cause mortality was estimated by

(2b) $$\text{RR}=\exp\left[\text{β}\left(\text{X}-{\text{X}}_{\text{0}}\right)\right]$$

X = mean concentration of the pollutants; X_o_ **=** baseline concentration: 10 μg/m^3^ for PM_10_; β = coefficient of risk function for short-term exposure (0.0008; 95% CI; 0.0006–0.0010) for all-cause mortality (Ostro, 2004). The choice of Ostro (2004) functions was based on their suitability for numerical application (Burnett et al., 2014). Attributable deaths (number of deaths attributable to exposure) could not be estimated due to lack of information about total number of deaths in the target population. It must be stated that levels of 10 μg/m^3^ and 3 μg/m^3^ are the counterfactual values above which health effects are normally calculated (Chalvatzaki et al., 2019). Other approach adopted involves the estimation of the magnitude of lifetime exposure to fine particles (in this case PM_2.5_ by investigating dose response assessment as described by EPA (1997)) from where excess lifetime cancer risk could be evaluated (Hyungkeun et al., 2018). The lifetime average daily dose (LADD) is given by Eq. (3) (EPA, 1997; Hyungkeun et al., 2018)

(3) LADD=(C×IR×EF×ED)/(BW×AT)

Combination of Eqs. (4) and (5) give the required excess lifetime cancer risk (ELCR) (Hyungkeun et al., 2018)

(4) slope factor,SF=UR/(BWxIR)

(5) ELCR=SF×LADD

UR = unit risk; C = pollutant's concentration (μg/m^3^); IR = inhalation rate (m^3^/day);
EF = exposure frequency (no unit) and BW = body weight (kg). In this study, 14.25 m^3^/day was used as inhalation rate which is mean value of 15.7 m^3^/day inhalation rate for adult males and 12.8 m^3^/day inhalation rate for adult females (Hyungkeun et al., 2018). Also, average weight of adults male and females was taken as 62.8 kg. Exposure duration was taken as 8 h (period of operation at the abattoir). 70 years was taken as the average time applied in cancer risk assessment as stipulated by U.S. EPA (2009) 0.008 μg/m^3^ chosen as unit risk value, based on the research by Greene and Morris (2006) was due to lack of information on slope factor in Nigeria, hence the need to calculate slope factor.

Also, health risk assessment of H_2_S, SO_2_ and NH_3_ was carried out by employing the method described by Gilbert et al. (2019). The non-carcinogenic risks induced by the inhalation of H_2_S, SO_2_ and NH_3_ were evaluated by calculating the hazard quotient (HQ) using Eq. (6) (U S EPA. 2009)

(6) $$\text{HQ}=\frac{\text{EC}}{\text{MRL}}$$

EC = exposure concentration (*μ*g/m^3^) and MRL = minimal risk level (*μ*g/m^3^). For acute exposures (exposure lasting 24 h or less), EC = CA (U S EPA, 2009), where CA = contaminant concentration in air (*μ*g/m^3^). Hence, Eq. (6) becomes,

(7) $$\text{HQ}=\frac{\text{CA}}{\text{MRL}}$$

Values of other parameters of Eqs. (1), (2a), (2b), (3), (4), (5), (6), and (7) are presented in Table 1.

**Table 1:** Health risk assessment input data.

Parameters	Value	Reference
coefficient of risk function,β	0.15515 (cardiopulmonary mortality)	Ostro (2004), Chalvatzaki et al.(2019)
	0.23218 (lung cancer mortality)	
	0.0008 (for all-cause mortality)	
Baseline concentration, X_0_	10 μg/m^3^ (PM_10_)	Ostro (2004), Chalvatzaki et al.(2019)
	3 μg/m^3^ (PM_2.5_)	
Inhalation rate	14.25 m^3^/day	Hyungkeun et al. (2018)
Exposure Duration	8 h	Hyungkeun et al. (2018)
Body weight	62.8 kg	Hyungkeun et al. (2018)
Unit risk (PM_2.5_)	0.008 μg/m^3^	Greene and Morris (2006)
Exposure frequency	0.85	EPA (1997)
Average time	70 years	US EPA (2009)
Minimum risk levels		
H_2_S	0.07 ppm (98 *μ*g/m^3^)	ATSDR, 2014
SO_2_	0.01 ppm (26.2 *μ*g/m^3^)	ATSDR, 1998
NH_3_	1.7 ppm	ATSDR, 2004

## 3 Results and discussion

### 3.1 Concentration of the pollutants and impacts on air quality

**Figure 2:**
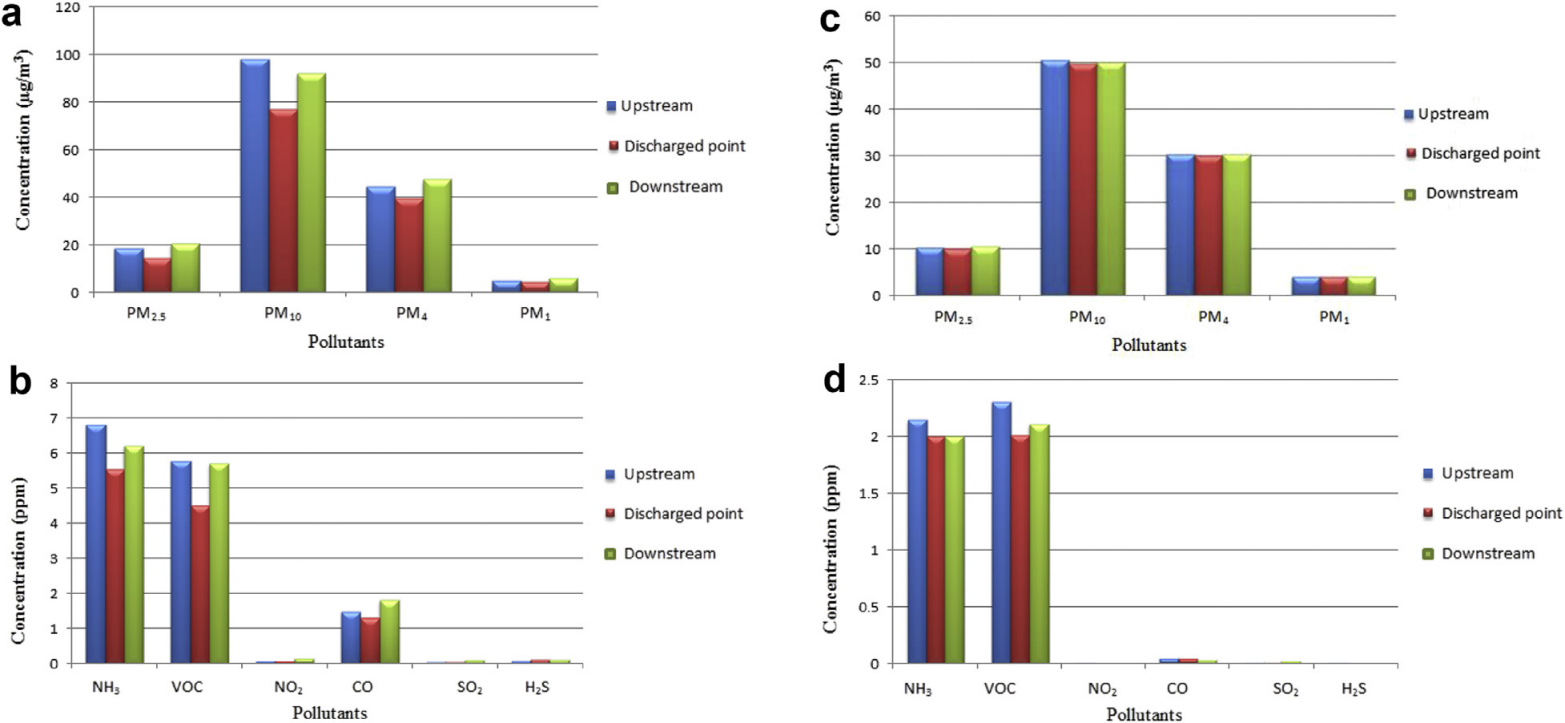
a: Particulate matter concentrations during workdays. b: Gaseous pollutants concentrations during workdays. c: Particulate matter concentrations during non-workdays. d: Gaseous pollutants concentration during non-work days (Saturdays and Sundays).

The mean concentrations of the pollutants at each sampling points during work days and non-work days at the abattoir were presented in Figure 2(a-d). During work days, except for PM_4_ which showed highest level of 47.57 ± 11.20 μg/m^3^ at upstream (sampling location A) while the lowest value of 39.63 ± 10.53 μg/m^3^ was recorded at the discharged point (sampling location B), other fractions of particulate showed highest concentration at the downstream and lowest at the discharged point (Figure 2a). Particulate matters, especially PM_2.5_ are thought to have originated from the animal fur being burnt. Similar situation was observed for gaseous pollutants. NH_3_ and VOC have highest mean concentrations at the upstream, while NO_2_, CO, SO_2_, and H_2_S showed highest concentrations at the downstream. As shown in Figure 2b, for all gaseous pollutants measured, lowest mean concentrations were recorded at the discharged point (though SO_2_ has concentrations of 0.05 ± 0.03 ppm at the discharged point being the same as the downstream concentration). Concentrations measured when the abattoir was not in operation were much lower than what was recorded during work days as depicted in Figure 2(c-d). This is an indication of the abattoir operation contribute in no small measure to the ambient in air quality of the host community.

Generally, low concentrations observed at the burning point (discharged location) as well as the high values recorded at distance from the discharged location could be linked to the pollutants being dispersed from emission source away to nearby locations. This observation is in consonance with the law of diffusion of gases, which explains the movement of gases from locations of elevated concentration to regions of reduced concentration. For H_2_S, downstream concentration of 0.10 ppm was found to be close to discharged location concentration (0.12 ppm). The variation in the mean concentration could be attributed to temperature, wind speed and wind pattern which are capable of influencing dispersion of atmospheric air (Magaji and Hassan, 2017). CO whose source is traceable to the kerosene being used for the burning of the animals has mean concentrations ranging from 1.30 ppm at the point of burning to 1.81 ppm downstream. It is revealed that for all pollutants, It is apparently clear that reduced concentration levels were observed at the point of release (discharged point) compared to the other two points. Although, at 95% confidence interval, no statistically significant difference was obtained between concentrations at downstream and upstream spots (p > 0.05); there is a meaningful statistically significant difference between concentrations at the discharged point (point B) and the other two points (p < 0.05). During non-work days, concentrations of the pollutants were much lower than what was recorded during abattoir operation (Figure 2b). PM_2.5_ PM_10_, PM_4_, PM_1_ have highest concentrations of 10.45 ± 4.72 μg/m^3^ (downstream), 50.65 ± 12.30 μg/m^3^ (upstream), 30.35 ± 6.83 μg/m^3^ (downstream) and 4.14 ± .1.23 μg/m^3^ (downstream), respectively. NO_2_, SO_2_ and H_2_S were almost undetectable. Apart from this, concentrations of individual pollutants at each point were found to be comparable as no meaningful statistically significant difference was obtained (p > 0.05).

Concisely, Table 2 showed the contribution of the abattoir operation to the ambient air quality of the environment in comparison with the recommended limits. While the mean concentration of PM_2.5_ which of 18.75 μg/m^3^ exceeded the limits recommended by WHO (10 μg/m^3^), the mean value of 89.17 μg/m^3^ for PM_10_ exceeded guidelines by WHO (10 μg/m^3^), FMEnv (50 μg/m^3^) and NAAQS (150 μg/m^3^). These constitute serious risk factors for public health (FEPA, 1999). The potential of particulate matter to cause health problem is a function of its size (FEPA, 1999). The mean concentrations of the pollutants recorded in this study were higher than the result reported by Magaji and Hassan (2017) but lower than the documented report of Ubuoh et al. (2017) Particulate matters whose size is less than 10 μm have been reported to have potential of causing health challenge of great magnitude because particles of this size can easily found their ways not only into the lung, but also into the blood streams which can result into lung and heart problems (Ubuoh et al., 2017). The mean value of NO_2_ (0.10ppm) also exceeded 0.02ppm, 0.03ppm and 0.05ppm limits recommended by WHO, FMEnv and NAAQS respectively. This is an indication that at frequent exposure, public health is at risk. Adverse respiratory symptoms in persons with asthma have been reported to be associated with the short term exposure to NO_2_ ranging from 30minutes to 24 h (US EPA, 2011). Mean concentration of SO_2_ is 0.07 ppm; while this value is below WHO recommended limit (0.08ppm), it exceeds both FMEnv and NAAQS recommended limits of 0.03 ppm. This suggests that a prolonged release of SO_2_ within the area is capable of worsening the health situation of people living with existing heart or respiratory issues (Ubuoh et al., 2017).

**Table 2:** Statistical distribution of the air pollutants and the recommended limits.

	PM_2.5_ (μg/m^3^)	PM_10_ (μg/m^3^)	PM_4_ (μg/m^3^)	PM_1_ (μg/m^3^)	NH_3_ (ppm)	VOC (ppm)	NO_2_ (ppm)	CO (ppm)	SO_2_ (ppm)	H_2_S (ppm)
Mean	18.57	89.17	43.99	5.25	6.19	5.33	0.10	1.52	0.07	0.10
SD	6.96	15.15	12.23	1.99	0.96	2.08	0.15	1.17	0.04	0.14
Max	43.05	108.20	72.55	9.30	8.00	7.95	0.40	3.65	0.2	0.6
Min	11.60	52.00	29.40	1.75	4.25	0.45	0.00	0.00	0.00	0.00
aWHO standard	25	50	-	-	-	-	0.02	25∗	0.08	
bFMENV standard	-	150	-	-	-	-	0.03	10∗∗	0.03	
cNAAQS	-	150	-	-	-	-	0.05		0.03	0.005

∗ 1 h mean

∗∗ 24 h mean.

a WHO (2000).

b FEPA (1991).

c FEPA (1999)

Air quality index (AQI) was also employed to further assess the impact of the abattoir on ambient air quality (Table 3). This index explains scale of rating for outdoor air. Low value of the scale is an indication of friendly air quality. Pollutants in ambient air are categorized into very good (0–15), good (16–31), moderate (32–49), poor (50–99) and very poor (greater than or equal to 100) with rating A, B, C, D and E respectively. Different priority gases have concentration limit which defines the category such pollutants will belong on the index. From the results, it was found that the ambient air quality in respect of CO and NH_3_ is very good, moderate for PM_10_ and is very poor for NO_2_ and SO_2_ (Table 4). This implies that apart from NO_2_ and SO_2_, all others were found to be within acceptable range. These results is in consonance with the results reported by Magaji and Hassan (2017) where only SO_2_ and NO_2_ were found to be in category E.

**Table 3:** Air Quality Index (AQI) for priority pollutants.

Category	Rating	PM_10_ (μg/m^3^)	CO (ppm)	NO_2_ (ppm)	SO_2_ (ppm)	NH_3_ (ppm)
Very good (0–15)	A	0–15	0–2	0–0.002	0–0.002	0–50
Good (16–31)	B	51–75	2.1–4	0.02–0.03	0.02–0.03	0–50
Moderate (32–49)	C	76–100	4.1–6.0	0.03–0.04	0.03–0.04	51–100
Poor (50–99)	D	101–150	6.1–9.0	0.04–0.06	0.04–0.06	201–300
Very poor (>100)	E	>150	>9.0	>0.06	>0.06	301–500

Source: US EPA, 2011.

**Table 4:** Air Quality Index of the analyzed pollutants.

Pollutants	AQI rating
PM_10_ (μg/m3)	Moderate (C)
CO(ppm)	Very good (A)
NO_2_ (ppm)	Very poor (E)
SO_2_ (ppm)	Very poor (E)
NH_3_ (ppm)	Very good (A)

### 3.2 Health risk assessment

Furthermore, the health risk assessment based on the estimated excess risk, attributable fractions, lifetime average daily dose and excess cancer lifetime risk for PM_10_ and PM_2.5_ as well as non-cancer risks of H_2_S, SO_2_ and NH_3_ are presented in Table 5. The results showed that for PM_2.5_, the highest excess risk of 0.48% was obtained for lung cancer at a distance of 100 m away (downstream) from the point of burning (point B) and 0.45% at a distance of 100m (upstream) before the burning location. This implies that there is at least 0.45% likelihood that an individual in a group of people exposed to PM_2.5_ 100 m away from the burning point will have health issue (lung cancer) than an individual from another set of people that is exposed to baseline concentration of 3 μg/m^3^. On the other hand, there is at least 0.37% chance that an individual in a group of people exposed to PM_2.5_ at the discharged point will have lung cancer. Similarly, highest excess risk of 0.30 % was obtained for cardiopulmonary mortality at the downstream while excess risk of 0.23% at the discharged point suggests a safer ambient PM_2.5_ concentration for an individual exposed to this pollutant under the same condition. For PM_10_, an individual from a set of group that stays 100 m before the discharged point has about 0.18% chance of having all-cause mortality higher than individual who is exposed to baseline concentration of10 μg/m^3^.

**Table 5:** Health risk assessment result.

Sampling locations		Upstream (A)	Discharged point (B)	Downstream (C)
**PM**_**10**_: **all-cause mortality, %**	ER	0.18	0.13	0.16
	AF	0.15	0.16	0.14
**PM**_**2.5**:_**Cardiopulmonary mortality, %**	ER	0.28	0.23	0.30
	AF	0.22	0.19	0.23
**PM**_**2.5**_**: Lung cancer mortality**	ER	0.45	0.37	0.48
	AF	0.31	0.27	0.32

SF = UR/(BW XIR) = 8.9 × 10^−6^ (For PM_2.5_)				
**LADD**		3.60	2.77	3.97
**ELCR**		3.20 × 10^−5^	2.47 × 10^−5^	3.53 × 10^−5^

Non-carcinogenic risks associated with the H_2_S, SO_2_ and NH_3_ via inhalation				
H_2_S		1.20	1.71	1.43
SO_2_		5.00	5.00	9.00
NH_3_		4.01	3.25	3.65

For attributable fraction, on the highest, 0.32% of deaths from lung cancer, and 0.23% from cardiopulmonary could be avoided if PM_2.5_ is reduced to 3 μg/m^3^ and while about 0.14% of all-cause mortality could be avoided if PM_10_ is reduced to of10 μg/m^3^. Further analysis to ascertain the significant difference between the excess risk and attributable fraction revealed that there is statistically meaningful significant difference between excess risk and attributable fraction (p < 0.05) for all the criteria pollutants. A similar findings by Arranz et al. (2014) revealed that 2% of all-cause mortality was connected to exposure to particulate matter. It was also reported that higher proportion of cardiopulmonary mortality could be avoided Chalvatzaki et al. (2019). Lifetime average daily dose (LADD) and excess lifetime cancer risk (ELCR) showed that individual at the discharged point will have lesser risk of cancer than persons 100m away from the point of discharge. In particular, cancer risk increased by about 30 % at upstream and 42% at the downstream. This is thought to be connected to diffusion of the pollutants from the point of release to the immediate environment. It was observed that there is no statistically significant difference between health risks at the downstream and at the upstream (p > 0.05). This implies that, irrespective of the location (either 100 m before of 100 m after) close to the abattoir in 100 m distance, the health risks remain the same.

The non-carcinogenic risks associated with the H_2_S, SO_2_ and NH_3_ via inhalation as evaluated by hazard quotient are also presented in Table 5. Generally, The HQ values below 1.0 indicate that the pollutants under investigation are not likely to cause health impairment, whereas HQ values above 1.0 indicate risk levels that are likely to damage health (Kitwattanavong et al., 2013; Gilbert et al., 2019). The HQ values at the three points for H_2_S are 1.20, 1.71 and 1.43 respectively, that of SO_2_ are 5.00, 5.00 and 9.00 respectively; while that of NH_3_ are 4.01, 3.25 and 3.65 respectively. All the HQ values exceeded the threshold value, set at the unity, implying that H_2_S, SO_2_ and NH_3_ are likely to cause adverse health effects in the area under study for now. Previous studies have reported similar findings (Chung et al., 2017; Ferrero et al., 2017; Sunisa et al., 2019). However, this submission is in contrast to the findings where exposure of individuals in the vicinity of landfill site in Cameroon and students in the vicinities of coal mines in South Africa will likely negligible no health threat (Gilbert et al., 2019; Olufemi et al., 2019).

## 4 Study limitation

Impacts of abattoir on ambient air quality and the associated health risks were assessed in this study. However, due to realistic constraints, there were identified limitations in terms of the research process. The main focus of the study was to evaluate the impact of the abattoir at some distances away from the operation point. Ideally, it is expected that exposure to the abattoir emission be measure at various distance from the discharged point but measurements could not be made further than 100 m (The closest receptor to the abattoir) from the discharged point due to uncooperative altitude of the residents around the abattoir. Also the choice of exposure duration of 8 h was based on the abattoir operators’ working hours. In reality individual exposure duration may vary depending on schedule and prevailing circumstances. Although, the adjoining road is about 200 m to the abattoir facility and thus traffic-related pollutants were not captured in this study, in reality, contribution from traffic-related air pollutants cannot be totally neglected. Also, the health risks might have been underestimated because only the concentrations of PM_10_, PM_2.5_, H_2_S, SO_2_ and NH_3_ were considered for the health risk assessment. The contributions of other pollutants especially volatile toxic compounds could be significant to health risk assessment. Furthermore, only exposure via inhalation was considered although, exposure through ingestion and skin absorption may occur, even lower concentration (Gilbert et al., 2019).

## 5 Conclusion

The study assessed the environmental impact and health risk associated with exposure to emission from abattoir. The results indicated that abattoir operation negatively impact ambient air quality and that location at the point of release of the emission are less impacted that locations 100 m away and thus lesser risk of lung cancer and cardiopulmonary challenges may be experienced by individuals at the point of discharged than individuals at a distance of 100 m. It is therefore concluded that continuous operation of this abattoir within the residential area can constitute a great environmental menace to the residents of the area and can result in complication to those with existing health challenge. It is therefore recommended that the abattoir be moved to a designated area outside residential vicinity where the effects will be minimal. This step would reduce the dispersion of pollutants to residential areas and also prevent human's exposure to offensive odour emanating from abattoir facility. Also, proper waste management system as well frequent and proper monitoring of the activities of the operations of the abattoir by the Environmental Protection Agencies are advocated.

## Declarations

### Author contribution statement

Ebenezer Leke Odekanle: Performed the experiments; Analyzed and interpreted the data; Wrote the paper.

Omowonuola Olubukola Sonibare, Funso Alaba Akeredolu: Conceived and designed the experiments; Contributed reagents, materials, analysis tools or data.

Johnson Oludare Odejobi, Bamidele Sunday Fakinle: Analyzed and interpreted the data; Contributed reagents, materials, analysis tools or data.

### Funding statement

This research did not receive any specific grant from funding agencies in the public, commercial, or not-for-profit sectors.

### Competing interest statement

The authors declare no conflict of interest.

### Additional information

No additional information is available for this paper.
